# Preserved intrinsic neural timescale organization with hierarchical variation in autism spectrum disorder

**DOI:** 10.1093/cercor/bhag121

**Published:** 2026-09-07

**Authors:** Yumi Shikauchi, Ryuta Aoki, Takashi Itahashi, Masaaki Shimizu, Taiga Naoe, Tsukasa Okimura, Haruhisa Ohta, Ryu-ichiro Hashimoto, Motoaki Nakamura

**Affiliations:** Medical Institute of Developmental Disabilities Research, Showa Medical University, Tokyo 157-8577, Japan; Medical Institute of Developmental Disabilities Research, Showa Medical University, Tokyo 157-8577, Japan; Graduate School of Medicine, Human Brain Research Center, Kyoto University, Kyoto 606-8507, Japan; Department of Language Sciences, Graduate School of Humanities, Tokyo Metropolitan University, Tokyo 192-0397, Japan; Medical Institute of Developmental Disabilities Research, Showa Medical University, Tokyo 157-8577, Japan; Graduate School of Medical and Dental Sciences, Institute of Science Tokyo, Tokyo 113-8510, Japan; Medical Institute of Developmental Disabilities Research, Showa Medical University, Tokyo 157-8577, Japan; Medical Institute of Developmental Disabilities Research, Showa Medical University, Tokyo 157-8577, Japan; Medical Institute of Developmental Disabilities Research, Showa Medical University, Tokyo 157-8577, Japan; Medical Institute of Developmental Disabilities Research, Showa Medical University, Tokyo 157-8577, Japan; Department of Language Sciences, Graduate School of Humanities, Tokyo Metropolitan University, Tokyo 192-0397, Japan; Medical Institute of Developmental Disabilities Research, Showa Medical University, Tokyo 157-8577, Japan

**Keywords:** autism spectrum disorder, inter-individual variability, intrinsic neural timescale, sensory processing, temporal hierarchy

## Abstract

Intrinsic neural timescales (INTs) index the temporal decay of neural activity and form a hierarchy from fast sensorimotor to slow transmodal regions. Although altered INTs have been reported in autism spectrum disorder (ASD), it remains unclear whether this hierarchical organization is preserved and how individual variability along it relates to sensory traits. Using resting-state fMRI from 182 participants (67 ASD, 115 typically developed controls [TDC]), we estimated INTs from the autocorrelation half-life and averaged them across four runs. The cortical INT hierarchy was preserved in ASD, with comparable sensorimotor-to-transmodal organization across groups. Regions operating at longer timescales exhibited relative prolongation in ASD, with effect size increasing along the hierarchy. However, no statistically significant group differences were identified at any spatial resolution examined. To characterize individual INT profiles relative to a TDC-derived template, we decomposed each participant’s profile into global shift, hierarchical scaling, and residual deviation components. Global shifts and hierarchical scaling were associated with demographic variation (primarily sex) rather than diagnosis, whereas residual deviations showed a modest association with sensory traits characterized by reduced sensory registration. These findings indicate that large-scale temporal organization is largely preserved in ASD, while individual-specific deviations from this hierarchy may underlie variability in sensory experience.

**Significance statement**

Intrinsic neural timescales (INTs) reflect how long neural activity persists over time and are organized across the cortex from fast sensorimotor to slower association regions. Altered INTs have been reported in autism spectrum disorder (ASD), but it remains unclear whether this large-scale temporal organization is fundamentally disrupted. Using multi-run resting-state fMRI, we show that the cortical organization of INTs is largely preserved in ASD. Instead of focal abnormalities, variation emerges as systematic shifts along the cortical hierarchy and as individual-specific deviations from the typical INT organization. Importantly, these deviations are modestly associated with sensory traits characterized by reduced sensory registration. These findings suggest that individual differences in cortical temporal dynamics may contribute to variability in sensory experience.

## Introduction

**Figure 1:**
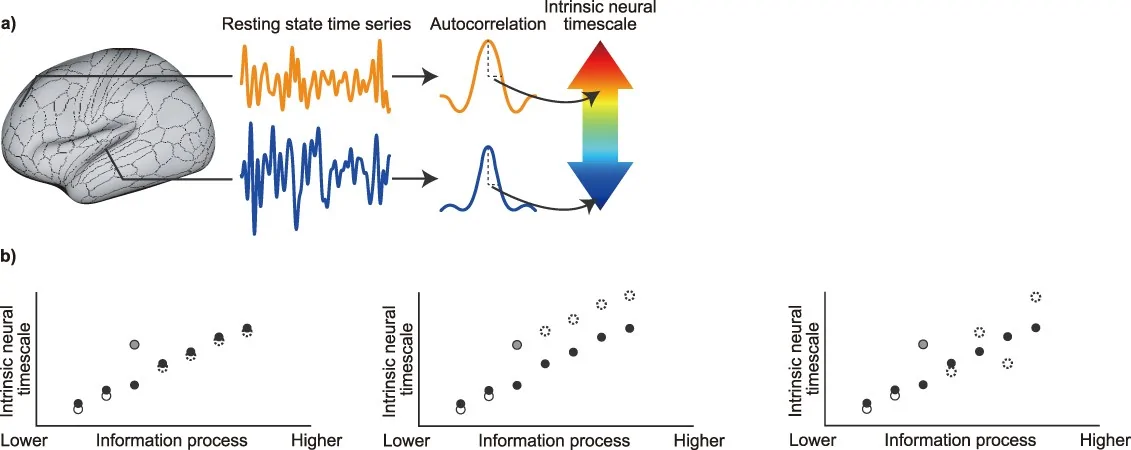
Conceptual illustration of the cortical hierarchy of intrinsic neural timescales (INTs) and its individual-level deviations. (a) INT was computed from resting-state fMRI time series as the first temporal lag at which the autocorrelation function decayed to 0.5, representing the characteristic temporal span over which spontaneous neural activity remains correlated with itself. Regions with longer INTs (orange) exhibit more slowly decaying autocorrelation, whereas regions with shorter INTs (blue) show faster signal fluctuations. INT provides a quantitative summary of the temporal persistence of neural activity at each cortical region and is commonly used to characterize large-scale temporal hierarchies across the cortex. (b) Solid black circles represent the typical INT profile, reflecting the orderly increase of timescales along the cortical processing hierarchy. Open circles indicate the INT values of a given individual. A gray dashed circle highlights a local deviation at a specific region. Three possible patterns of INT modulation are illustrated. Left: A focal deviation confined to a single region, with no systematic propagation to other hierarchical levels. Center: A cascading deviation in which downstream regions along the information-processing hierarchy continue to shift in the same direction, preserving hierarchical order but altering its global scaling. Right: A destabilized pattern in which downstream regions exhibit irregular or non-monotonic deviations, suggesting disruption of hierarchical organization.

The human brain processes information across multiple temporal scales, forming a hierarchical organization that mirrors its anatomical and functional architecture (Honey et al. 2012; Ito et al. 2020; Golesorkhi et al. 2021). The intrinsic neural timescale (INT) has been proposed as one of several indices to describe temporal hierarchy, indicating the duration over which neural activity remains autocorrelated within a region (Fig. 1a) (Murray et al. 2014). Neural activity in primary sensorimotor areas fluctuates rapidly, reflecting short INT, whereas association cortices integrate information over longer timescales, supporting abstraction, prediction, and self-referential processing. This gradient of temporal integration, observed across sensorimotor-to-association hierarchies and conserved across mammalian cortices (Murray et al. 2014; Runyan et al. 2017), is considered a fundamental principle of cortical computation that enables the coordination of perception and cognition over time. Additionally, although INT was originally introduced using electrocorticographic recordings, subsequent studies have extended the framework to electroencephalography and functional magnetic resonance imaging (fMRI), with evidence suggesting that its hierarchical organization is broadly preserved across measurement modalities (Watanabe et al. 2019).

Individuals with autism spectrum disorder (ASD) exhibit atypical sensory experiences and difficulties in integrating and predicting information over time, suggesting alterations in both temporal and hierarchical organization of neural processing. Prior neuroimaging studies have reported regional alterations of INTs in ASD (Watanabe et al. 2019; Uscătescu et al. 2023), yet the reported effects have varied considerably in both direction and anatomical location. Such inconsistencies suggest that ASD-related differences in INTs may not be adequately described by region-wise comparisons alone, as these approaches do not account for inter-individual variation in global INT baselines or hierarchical scaling across the cortex. Consequently, local deviations may reflect shifts in the relative positioning of regions within the cortical hierarchy rather than isolated abnormalities (Fig. 1b). This perspective motivated the hypothesis that ASD involves system-level reorganization of cortical timescale hierarchy, rather than focal disruptions in specific regions (Bernhardt et al. 2025). Gradient-based and network-level analyses indicate a reduced differentiation between sensorimotor and transmodal systems, suggesting that ASD may involve broad hierarchical modulation rather than focal dysfunction. This hierarchical imbalance could provide a unifying framework for understanding both enhanced perceptual sensitivity and difficulties in higher-order integration observed in ASD.

From a theoretical perspective, alterations in INT are unlikely to remain confined to isolated cortical loci (Murray et al. 2014; Chaudhuri et al. 2015). Because each region’s temporal integration depends on interactions with other levels of the cortical hierarchy, a local perturbation of timescales would disrupt the coordinated flow of information across the system. Therefore, deviations in INT are expected to be accompanied by distributed, hierarchical adjustments that preserve large-scale coherence in neural processing. Building on this framework, the present study aimed to examine whether ASD involves alterations in the large-scale organization of INT rather than focal abnormalities restricted to specific cortical regions. We analyzed resting-state fMRI data from a large cohort collected through the Brain/MINDS Beyond project (Koike et al. 2021), allowing for a comprehensive assessment of the cortical hierarchy in both ASD and typically developed controls (TDC). In addition, by relating INT to individual differences in sensory processing profiles, we sought to determine whether the association between neural timescales and sensory traits differs across diagnostic groups.

Based on these considerations, we examined whether ASD-related alterations in INT are better characterized as local changes confined to specific sensorimotor regions or as more globally distributed modulations across the cortical hierarchy. To place these temporal hierarchy patterns in a broader biological context, we also compared INT with microstructural indices, including T1w/T2w signal intensity maps (Glasser and Van Essen 2011; Burt et al. 2018) and neurite density index (NDI) derived from diffusion-weighted imaging (Zhang et al. 2012; Fukutomi et al. 2018). These multimodal comparisons allow us to examine whether the large-scale organization of temporal processing aligns with, or departs from, underlying structural gradients previously linked to temporal features of brain activity (Shafiei et al. 2023). Furthermore, by examining how individual variability along the cortical INT hierarchy relates to sensory processing profiles, we sought to clarify whether deviations from large-scale temporal organization are associated with sensory atypicalities in ASD.

## Materials and methods

### Experimental design

#### Participants

Data were collected as a part of Brain/MINDS Beyond projects (Koike et al. 2021), which established a high-quality scanning protocol, the Harmonization Protocol (HARP) to acquire multimodal brain images including T1- and T2-weighted, resting-state and task-based functional, and diffusion-weighted MRI. The present study analyzed resting-state data from 316 participants who met all inclusion criteria as follows. To minimize potential disparities introduced by variations in imaging equipment, only data acquired using the Skyra fit scanner were included. Participants were required to be labeled as either TDC or individuals with ASD, to have completed four resting-state fMRI runs, and to have fully responded to the Adolescent/Adult Sensory Profile (AASP) (Brown et al. 2001). Participants were excluded if they had any structural brain anomalies, were younger than 18 years, exhibited severe motion or imaging artifacts, had mean framewise displacement (FD) exceeding three standard deviations from the overall mean (FD > 0.178 mm). A clinical diagnosis of ASD was required for inclusion in the ASD group; individuals with neurodevelopmental diagnoses in the absence of ASD (e.g., Attention-Deficit/Hyperactivity Disorder (ADHD)-only) were excluded. Other psychiatric comorbidities were not systematically screened and therefore were not used as exclusion criteria. All participants were community-dwelling individuals capable of independently volunteering for MRI research. After applying these criteria, 115 TDC and 67 ASD participants remained for analysis (Table 1). Information regarding current medication use was collected. In the ASD group, three participants were taking methylphenidate (Concerta) and one was taking atomoxetine (Strattera), whereas no participants in the TDC group were receiving ADHD medication. Medication use was not used as an exclusion criterion. Estimated intelligence quotient (IQ) based on the Japanese adult reading test (JART) was reported for both groups. No TDC participants had JART scores below 85. The Wechsler Adult Intelligence Scale Fourth Edition (WAIS-IV) or Third Edition (WAIS-III) was administered in the ASD group. No ASD participants had both JART and WAIS-IV/WAIS-III scores below 85. IQ was therefore not used as an exclusion criterion. This study was approved by the institutional ethics committee of Showa Medical University (approval number: B-2014-019). All the participants provided written informed consent. All procedures were conducted according to the Declaration of Helsinki.

**Table 1:** Demographics of the analyzed population.

	# of participants	Age mean ± SD (min–max)	Framewise displacement mean ± SD (min–max)	Estimated IQ by JART mean ± SD (min–max)	IQ by WAIS IV mean ± SD (min–max)	IQ by WAIS III mean ± SD (min–max)
**ASD**	67 (Male 46, 69%)	27.925 ± 7.768 (18–48)	0.072 ± 0.027 (0.033–0.147)	105.653 ±7.743 (75.338–119.800)	102.609 ± 14.693 (75–131) (n = 41)	101.800 ± 11.020 (78–118) (n = 15)
**TDC**	115 (Male 88, 77%)	26.321 ± 6.375 (18–45)	0.063 ± 0.024 (0.025–0.148)	108.253 ± 7.743 (85.443–119.800)		

IQ, intelligence quotient; JART: Japanese adult reading test; WAIS, Wechsler Adult Intelligence Scale.

#### MRI data acquisition

All MRI data were acquired following the HARP implemented in the Brain/MINDS Beyond project (Koike et al. 2021). Scanning was performed on a 3T Siemens MAGNETOM Skyra fit scanner equipped with a 32-channel head coil.

Structural T1-weighted and T2-weighted images were obtained using a high-resolution 3D MPRAGE and 3D SPACE sequences (0.8-mm isotropic), respectively. Diffusion-weighted images were acquired using a spin-echo echo planar imaging (EPI) sequence with 1.7-mm isotropic resolution and a multishell diffusion scheme (b = 0, 700, and 2000 s/mm^2^). Diffusion weighting was applied along multiple gradient directions for each shell, with phase-encoding directions alternated between anterior-to-posterior and posterior-to-anterior across acquisitions. Resting-state fMRI data were collected using a multiband EPI sequence (TR = 800 ms; 2.4-mm isotropic). Each participant completed four runs of resting-state fMRI (5 min per run), with two runs acquired in the anterior-to-posterior direction and two in the posterior-to-anterior direction.

#### MRI data preprocessing

Preprocessing of the HARP data was performed using the Human Connectome Project (HCP) minimal preprocessing pipeline (Glasser et al., 2013). Briefly, this pipeline includes gradient distortion correction, motion correction, and EPI susceptibility-distortion correction using paired spin-echo EPI images with opposite phase-encoding directions, followed by registration to structural images and standard surface/volume spaces. Residual artifacts were further reduced using ICA-FIX denoising, applied in a multi-run manner to leverage the four resting-state runs per participant. The preprocessed fMRI data were then used for the estimation of INT maps in 32k Conte69 mesh surface space with native spatial resolution. The T1w/T2w signal intensity maps (Glasser and Van Essen 2011) in 32k Conte69 mesh surface space were used to compare functional (INT) and structural (T1w/T2w) measures of hierarchy. In addition, NDI maps derived from diffusion-weighted imaging were employed to assess microstructural hierarchy. Following Fukutomi et al. (2018), diffusion-weighted signals were fitted with a multi-compartment model, and the volume fraction of the intra-cellular compartment was used as NDI.These maps were also resampled to the 32k Conte69 mesh surface space for comparison with the functional and T1w/T2w measures.

### Statistical analyses

#### INT calculation

Before estimating the vertexwise INT values, preprocessed fMRI data were further proc- essed with the following steps: (1) regression of nuisance variables, including the top aCompCor components (five components each from white-matter and cerebrospinal-fluid masks), global-brain signal, and the 24 motion parameters along with their first derivatives, resulting in a total of 30 nuisance regressors (Behzadi et al. 2007; Itahashi et al. 2025); (2) bandpass filtering in the 0.01–0.1 Hz range; (3) excluding volumes with FD greater than 0.5 mm, as well as the volumes immediately preceding and following those volumes, from subsequent analyses (Power et al. 2012).

The calculation of the INT was conducted as proposed in a previous study (Raut et al. 2020). Briefly, for each cortical vertex, the autocorrelation function (ACF) of the resting-state time series was computed using lags from -20 to +20 TRs (32.8 s). INT was defined as the first temporal lag at which the ACF decayed to 0.5, reflecting the temporal persistence of neural activity. To estimate this decay point with finer temporal resolution than the acquisition TR, discrete ACF samples were spline-interpolated using the Shape Language Modeling toolbox (https://www.mathworks.com/matlabcentral/fileexchange/24443-slm-shape-language-modeling). Following motion censoring, ACFs were estimated only from temporally continuous segments containing at least 21 consecutive volumes, without treating censored time points as temporally contiguous samples (Raut et al. 2019).

The vertexwise INT values were then averaged across the four runs to obtain a stable estimate for each participant. To obtain parcel- and network-level measures, run-averaged vertexwise INT values were aggregated within Glasser’s multimodal cortical 360 parcels and subsequently within the 12 functional networks defined by the Cole–Anticevic Brain-wide Network Partition (Glasser et al. 2016; Ji et al. 2019).

#### Sensory hierarchy correspondence analysis

To examine whether participant-level INT profiles followed previously reported sensory hierarchies, we quantified the correspondence between regional INTs and predefined canonical sensory hierarchies within auditory, visual, and somatosensory systems described by Wengler et al. (2020). For each participant and sensory system, Spearman’s rank correlations were computed across cortical regions constituting the predefined hierarchy. The resulting participant-level correlation coefficients were compared between groups using linear models including diagnostic group, age, sex, and mean FD as predictors.

To further assess the consistency of hierarchical ordering across participants within each diagnostic group, we additionally calculated Kendall’s coefficient of concordance (*W*) across cortical regions within each sensory hierarchy. Group differences in concordance were evaluated using bootstrap resampling.

#### Structure–function correspondence analysis

To assess structure–function correspondence within sensory hierarchies, we quantified the spatial correspondence between regional INT profiles and microstructural measures, including T1w/T2w signal intensity and NDI. For each participant and within each sensory system, Spearman’s rank correlations were calculated across cortical regions constituting the predefined sensory hierarchy described by Wengler et al. (2020). The resulting participant-level correlation coefficients were used as indices of structure–function correspondence. Group differences in these indices were assessed using linear models including diagnostic group, age, sex, and mean FD as predictors.

To account for spatial autocorrelation in regional cortical maps, we additionally performed spin-based spatial null testing (Alexander-Bloch et al. 2018; Markello and Misic 2021). Glasser parcel centroids were projected onto the fs_LR spherical surface, randomly rotated, and reassigned to the nearest parcel within the same hemisphere (Vázquez-Rodríguez et al. 2019). For each sensory hierarchy, the set of parcels comprising the hierarchy was rotated on the spherical surface while preserving the spatial arrangement among parcels, and null distributions generated from 10,000 spin permutations. The observed correspondence between INT and microstructural measures was then compared against these null distributions. Spin-based *p*-values were calculated as the proportion of null correlations whose absolute values exceeded the observed correlation magnitude.

#### Large-scale spatial trend analysis

To evaluate whether ASD-related INT differences were systematically organized along the cortical timescale hierarchy, we quantified the association between regional INT values and the magnitude of group differences (Cohen’s *d* for TDC–ASD) across cortical vertices, parcels, and functional networks. Statistical significance was assessed using spin-based spatial null testing (10,000 permutations) at parcel and vertex levels. For parcel-wise analyses, the procedure described above was applied to Glasser parcel centroids. For vertex-wise analyses, rotations were performed directly on the fs_LR cortical surface.

#### Quantification of individual deviations relative to the canonical hierarchical ordering of INTs

To evaluate individual variability in INTs beyond global shifts and hierarchical scaling, we characterized subject-specific deviations relative to an empirically derived INT hierarchy.

To establish a reference for this canonical organization, an INT template was constructed by averaging parcel-wise INT values across TDC. This template defined an INT-based cortical hierarchy, against which individual INT profiles were evaluated.

For each participant *i*, parcel-wise INT values ${y}_{i,p}$ were modeled as a linear transformation of the template INT values ${t}_p$ using ordinary least squares regression:


(1) \begin{eqnarray*} {y}_{i,p}={\alpha}_i+{\beta}_i{t}_p+{r}_{i,p} \end{eqnarray*}

where ${\alpha}_i$ captures a subject-specific global offset in INTs, ${\beta}_i$ reflects the degree to which the individual INT profile follows the hierarchical gradient of the template, and ${r}_{i,p}$represents residual deviations not explained by linear alignment to the template. This formulation allows separation of overall INT prolongation or shortening and hierarchical scaling effects from non-linear, spatially distributed deviations. To avoid circularity, when computing ${t}_p$for participants in the TDC group, each participant’s own data was excluded from the TDC reference distribution used to derive the hierarchy.

Individual variability in INT distribution was quantified as the root mean square (RMS) of the residuals across all parcels:


$${\mathrm{rms}}_i=\sqrt{\frac{1}{360}\sum_{p=1}^{360}{r}_{i,p}^2,}$$

where ${r}_{i,p}$ were computed after linear alignment of individual INT profiles to the TDC-derived template. As the analysis was performed on a fixed parcellation comprising 360 cortical parcels, this metric provides a consistent measure of distributed deviations from the INT-based cortical hierarchy across participants.

To relate individual INT deviations to sensory traits, we analyzed associations between *rms* and sensory processing measures assessed by AASP. Given substantial correlations among the four AASP subscale scores (Turjeman-Levi and Kluger 2022), principal component analysis (PCA) was applied to derive orthogonal sensory dimensions. To assess relationships between individual INT deviations and sensory traits, we computed Spearman’s rank partial correlations between the residual RMS measure of INT and AASP principal component (PC) scores, while controlling for age, sex, and FD.

## Results

### INTs reflect sensory processing hierarchies

We estimated INTs from resting-state fMRI by quantifying the lag at which the autocorrelation function decayed to 0.5 for each cortical region (Fig. 1a) (Ito et al. 2020; Raut et al. 2020). To confirm whether the previously reported hierarchical organization of INTs was also present in individuals with ASD, we evaluated the correspondence between participant-level INT profiles and predefined sensory hierarchies within auditory, visual, and somatosensory processing systems. These sensory systems were selected because their hierarchical organization—from primary to higher-order association areas—has been systematically delineated in previous anatomical and functional studies, providing a well-defined canonical scaffold against which the cortical hierarchy of INTs can be evaluated.

**Figure 2:**
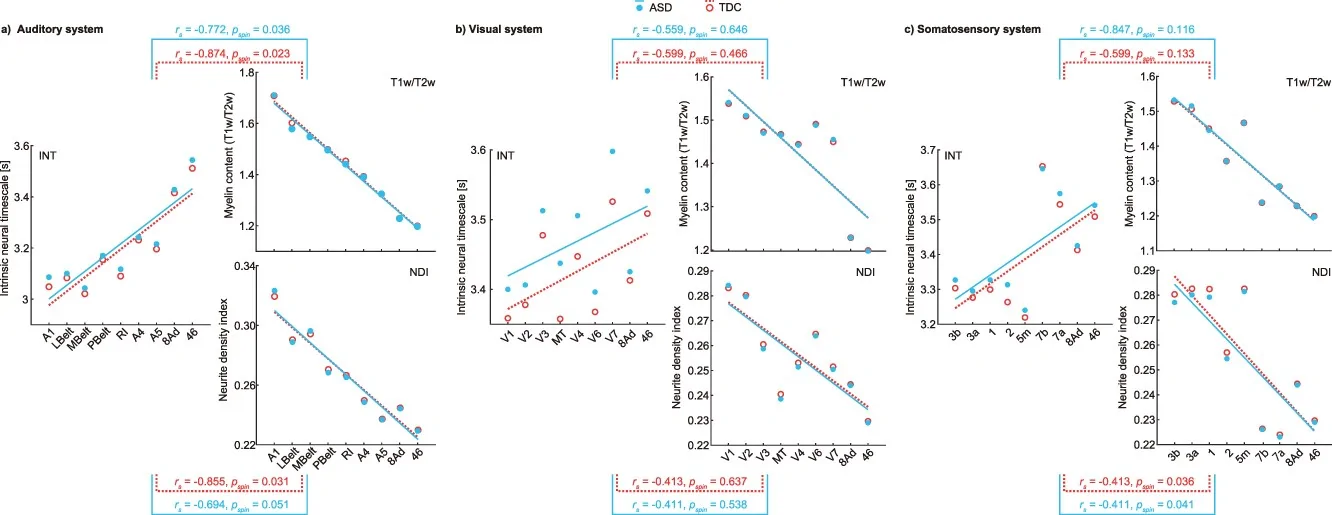
Correspondence between intrinsic neural timescales (INTs), predefined sensory hierarchies, and microstructural profiles. (a-c) Auditory (a), visual (b), and somatosensory (c) sensory hierarchies based on Wengler et al. (2020). Left panels show estimated marginal means of INTs across hierarchical levels in autism spectrum disorder (ASD; cyan) and typically developed controls (TDC; red), adjusted for age, sex, and framewise displacement (FD). Right panels show microstructural measures across hierarchical levels within each sensory system (upper panels, T1w/T2w signal intensity; neurite density index [NDI]). Partial correlation coefficients (*r_s_*), adjusted for age, sex, and FD, are shown together with *p*-values obtained from spin-based spatial null testing (*p_spin_*). Solid cyan lines indicate ASD; dotted red lines indicate TDC.

Within auditory processing regions, participant-level Spearman correlations between regional INTs and the predefined anatomical hierarchy described by Wengler et al. (2020) were consistently positive in both groups (median *r_s_* [IQR]: ASD = 0.850 [0.166]; TDC = 0.850 [0.145]) (Fig. 2a). Linear models controlling for age, sex, and FD confirmed that participant-level correlation coefficients were significantly greater than zero in both ASD (intercept estimate = 0.808, *p* < 1 × 10^−5^) and TDC (intercept estimate = 0.859, *p* < 1 × 10^−5^). None of the covariates (age, sex, FD) showed significant effects (all *p* > 0.400). No significant group difference was observed in the participant-level correlation coefficients (ANCOVA including age, sex, and FD as covariates, *F*(4, 177) = 0.804, *p* = 0.523). The main effect of group was not statistically significant (*t*(177) = 1.415, *p* = 0.158). The effect size was small (Cohen’s d ≈ 0.21), indicating that the auditory INT hierarchy was broadly comparable between ASD and TDC. Similar results were obtained when INTs were estimated using the alternative area-under-curve (AUC)-based definition proposed by Watanabe et al. (2019) (Supplementary Table S1).

We additionally quantified the consistency of hierarchical ordering across participants within each group using Kendall’s coefficient of concordance (*W*). Auditory hierarchies showed high concordance in both groups (TDC: *W* = 0.805; ASD: *W* = 0.720), indicating broadly preserved canonical ordering despite modestly reduced consistency in ASD. Bootstrap analyses further indicated that concordance was reliably lower in ASD than in TDC (95% bootstrap confidence interval (CI) for ASD–TDC difference: [-0.158, -0.009]), suggesting increased inter-individual variability in auditory hierarchical organization within the ASD group.

Similar results were observed in the visual and somatosensory processing hierarchies (Fig. 2b, c). In the visual system, participant-level Spearman correlations between regional INTs and the predefined histological hierarchy were positive in both groups (median *r_s_* [IQR]: ASD = 0.283 [0.566]; TDC = 0.333 [0.645]). Linear models controlling for age, sex, and FD confirmed that participant-level correlation coefficients were significantly greater than zero in both ASD (intercept estimate = 0.539, *p* = 0.017) and TDC (intercept estimate = 0.567*, p* = 0.004). No significant group difference was observed in the participant-level correlation coefficients (*p* = 0.307), and the estimated effects of age, sex, and FD were close to zero (all *p* > 0.32). Additionally, concordance across participants was relatively weak in both groups (TDC: *W* = 0.207; ASD: *W* = 0.238), suggesting that local reversals in hierarchical ordering may have limited alignment to the canonical hierarchy. Bootstrap analyses did not indicate a reliable group difference in concordance (95% bootstrap CI: [-0.049, 0.127]). In the somatosensory processing system, participant-level Spearman correlations between regional INTs and the histological hierarchy were positive in both groups (median *r_s_* [IQR]: ASD = 0.550 [0.408]; TDC = 0.567 [0.417]). Linear models controlling for age, sex, and FD confirmed that participant-level correlation coefficients were greater than zero in both ASD (intercept estimate = 0.744, *p* = 2 × 10^−4^) and TDC ( intercept estimate = 0.599, *p* = 3 × 10^−4^). No significant group difference was observed (*p* = 0.354). Estimated effects of age, sex, and FD were small (all p > 0.09). Concordance analyses indicated intermediate consistency in both groups (TDC: *W* = 0.556; ASD: *W* = 0.486), with a tendency toward reduced consistency in ASD, although bootstrap CI overlapped zero (95% bootstrap CI: [-0.195, 0.062]). Together, these findings indicate that correspondence to canonical sensory INT hierarchies is broadly preserved in ASD, while also suggesting modestly increased inter-individual variability in hierarchical organization, particularly within the auditory system.

Additional split-half analyses demonstrated moderate-to-high within-subject reliability of sensory INT hierarchies across independent run subsets in both ASD and TDC groups (Supplementary Methods; Supplementary Table S2).

### INT hierarchies align with cortical microstructural organization

To further validate the reliability of the temporal hierarchy captured by INT in individuals with ASD, we compared it with structural measures known to reflect cortical hierarchy. Specifically, we examined the relationships between INT, T1w/T2w signal intensity, and NDI, both of which have been established as structural proxies of hierarchical organization (Burt et al. 2018; Fukutomi et al. 2018; Huntenburg et al. 2018; Paquola et al. 2019). Within each individual, we computed individual-level pairwise correlations between regional INTs and T1w/T2w values (median Spearman’s *r_s_* [IQR]: ASD = -0.808 [0.217]; TDC = -0.833 [0.146]), as well as between INTs and NDI (ASD = -0.783 [0.221]; TDC = -0.817 [0.167]) for auditory system (Fig. 2a). To account for spatial autocorrelation in cortical maps, we additionally performed spin-based spatial null testing. The correspondence between INT and T1w/T2w within the auditory hierarchy remained significant at the group level after spin-based correction in both ASD (*p_spin_* = 0.036) and TDC (*p_spin_* = 0.023). The INT–NDI correspondence remained statistically significant after spin-based correction in the TDC group (*p_spin_* = 0.031), but not in the ASD group (*p_spin_* = 0.051). These findings indicate that the observed relationship between auditory INT hierarchy and cortical microstructural organization cannot be fully explained by spatial autocorrelation alone. Together, these results replicate the previously reported negative relationship between INTs and T1w/T2w values in healthy adults (Ito et al. 2020) and support the notion that the hierarchical gradient of INT is coupled with cortical microstructural organization.

Similar structure–function correspondence analyses were performed for the visual and somatosensory systems. In the visual hierarchy, participant-level INT–T1w/T2w correspondence was relatively weak in both groups (ASD = -0.267 [0.750]; TDC = -0.400 [0.662]), as was participant-level INT– NDI correspondence (ASD = -0.317 [0.608]; TDC = -0.267 [0.533]) (Fig. 2b). Consistent with these weak participant-level correspondences, spin-based spatial null testing did not indicate statistically significant correspondence for either T1w/T2w or NDI in either group (all *p_spin_* > 0.46). In the somatosensory system, INT demonstrated negative correlations with both T1w/T2w values (ASD = -0.633 [0.416]; TDC = -0.616 [0.412]) and NDI (ASD = -0.733 [0.279]; TDC = -0.750 [0.316]) (Fig. 2c). Under spin-based spatial null testing, the correspondence between INT and T1w/T2w was not statistically significant at the group level (ASD: *p_spin_* = 0.117; TDC: *p_spin_* = 0.133). In contrast, the relationship between INT and NDI showed statistically significant correspondence in both ASD (*p_spin_* = 0.041) and TDC (*p_spin_* = 0.037).

Together with the hierarchical orderings observed across sensory systems, these findings indicate that at least part of the association between sensory INT hierarchy and cortical microstructural organization cannot be fully explained by spatial autocorrelation alone.

### Preserved cortical hierarchy of INTs in ASD

**Figure 3:**
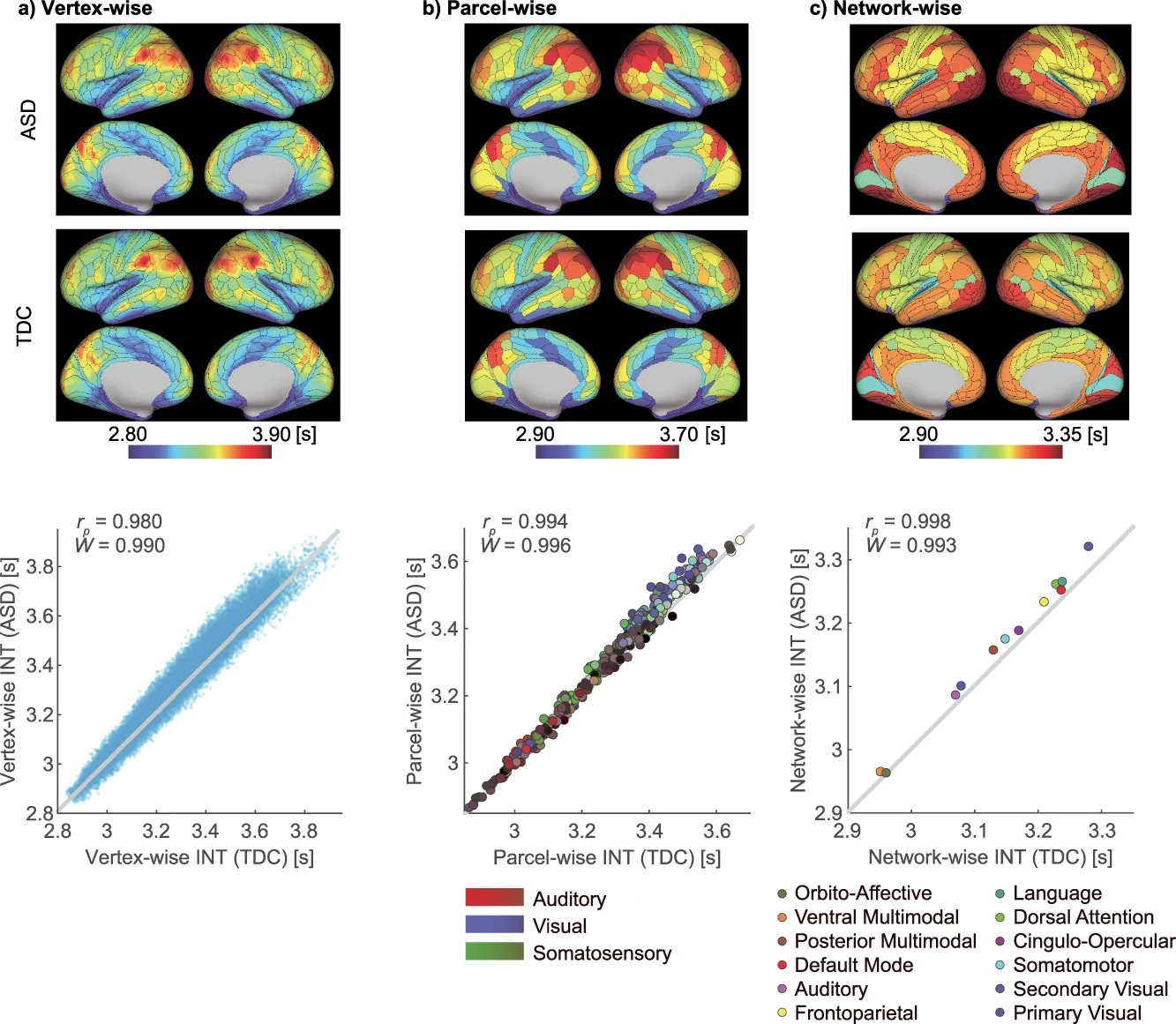
Preserved cortical hierarchy of intrinsic neural timescales (INTs) in autism spectrum disorder (ASD). Cortical surface maps show vertex- (a), parcel- (b), and network-wise (c) INTs in ASD (top) and typically developed controls (TDC; middle), together with the corresponding cross-group comparisons (bottom). Scatter plots illustrate the correspondence of INTs between groups at each spatial scale. Each dot represents a vertex, cortical parcel, or functional network, and the gray diagonal line indicates perfect correspondence (Pearson’s correlation coefficient *r_p_* = 1) between ASD and TDC. Across all levels—From fine-grained vertices to large-scale functional networks—INTs exhibited an almost identical spatial distribution between ASD and TDC (vertex-wise: Kendoll’s *W* = 0.990, *p* < 1×10^−5^; parcel-wise: *W* = 0.996, *p* < 1×10^−5^; network-wise: *W* = 0.993, *p* = 0.026). In panel (b), colors correspond to cortical regions defined in the Glasser multimodal parcellation (Glasser et al. 2016), whereas in panel (c), colors correspond to canonical large-scale functional networks based on the Cole–Anticevic brain-wide network partition (Ji et al. 2019).

Building upon the preserved sensory hierarchy described above, we next examined whether the large-scale cortical hierarchy of INTs is similarly maintained in individuals with ASD. Vertex-, parcel-, and network-wise INTs were estimated for both TDC and individuals with ASD (Fig. 3). Group-averaged INT maps were computed from values adjusted for age, sex, and mean FD. The preserved spatial correspondence was clearly visible in the cortical surface maps, which displayed consistent gradients of INTs from primary sensorimotor to higher-order association regions in both ASD and TDC. Longer INTs were observed in association cortices, such as the default mode and frontoparietal networks, whereas shorter INTs appeared in primary sensory and motor regions. This hierarchical gradient mirrors previous findings in neurotypical populations (Raut et al. 2020; Wengler et al. 2020) and suggests that the global organization of temporal integration is maintained in ASD.

To quantitatively assess this preservation, we compared the spatial correspondence of INTs between ASD and TDC across multiple scales. INTs exhibited remarkably similar spatial distributions between the two groups. Vertex-wise INTs showed extremely high concordance at the group level (Pearson’s correlation coefficient *r_p_* = 0.980, Kendall’s *W* = 0.990; both *p*-values < 1 × 10^−5^), indicating that both the absolute magnitude and the relative hierarchical order of cortical INTs were nearly identical between ASD and TDC. Comparable results were obtained at the parcel-wise scale (*r_p_* = 0.994, *W* = 0.996; both *p*-values < 1 × 10^−5^) and at the network-wise scale (*r_p_* = 0.998, p < 1 × 10^−5^; *W* = 0.993; *p* = 0.026), further confirming that the large-scale temporal hierarchy of the cortex is preserved in ASD. Additional robustness analyses, including split-half analyses using independent run subsets (all *r_p_* > 0.967; Supplementary Methods) and FD-matched sensitivity analyses (Supplementary Methods; Supplementary Fig. S1), yielded highly similar results across all spatial scales.

Taken together, these results demonstrate that, beyond modality-specific hierarchies such as the auditory system, the overall cortical hierarchy of INTs is robustly preserved in ASD.

### Large-scale spatial trends in group-related divergence

**Figure 4:**
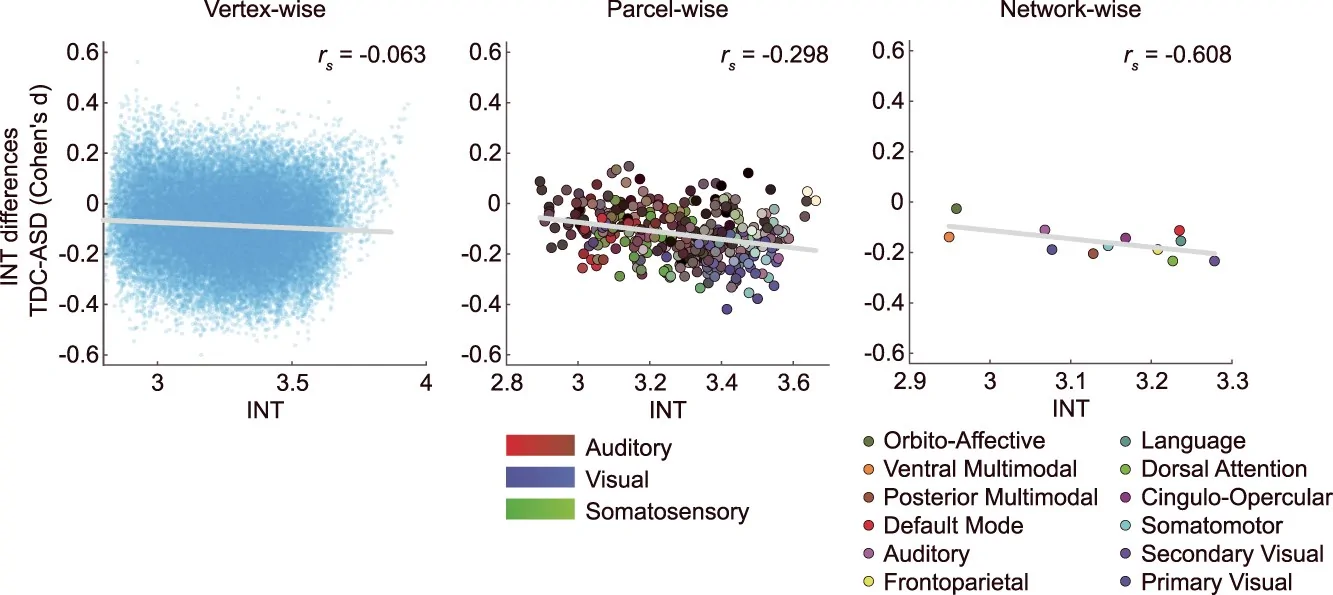
Large-scale spatial trends in ASD-related divergence along the cortical timescale hierarchy. Scatter plots show the relationship between intrinsic neural timescales (INTs) in TDC (*x*-axis) and the corresponding group-related divergence in INTs (Cohen’s *d* for TDC–ASD, *y*-axis) at the vertex (left), parcel (center), and network (right) levels. Each dot represents a cortical vertex, parcel, or network, respectively. The light gray line represents a fitted linear trend for visualization, whereas the numerical values displayed at the top of each panel indicate Spearman’s correlation coefficients (*r_s_*) quantifying the monotonic association between INTs and group differences. Colors in panel center correspond to cortical regions defined in the Glasser multimodal parcellation (Glasser et al. 2016), and colors in panel right correspond to canonical large-scale functional networks based on the Cole–Anticevic brain-wide network partition (Ji et al. 2019).

To further characterize how group differences vary across the preserved cortical hierarchy, we examined the relationship between the length of INTs and the magnitude of group differences (Cohen’s *d* for TDC–ASD) across multiple spatial scales (Fig. 4). All analyses were performed after adjusting for age, sex, and FD.

No regions showed statistically significant effects after Benjamini–Hochberg false discovery rate (BH-FDR) correction across regions at any spatial resolution (*p_FDR_* > 0.050), indicating that the present dataset did not provide statistically significant evidence for localized group differences. For transparency and descriptive purposes, parcels showing nominal *p*-values < 0.05 are summarized in Supplementary Table S3 and Figure S2. Nevertheless, a consistent trend was observed across multiple spatial resolutions: cortical regions with longer INTs tended to show progressively larger TDC–ASD differences, consistent with relatively prolonged INTs in ASD along the global cortical timescale hierarchy. This negative association was evident at the vertex (Spearman’s *r_s_* = –0.063), parcel (*r_s_* = –0.298), and network (*r_s_* = –0.608) levels, with effect sizes becoming larger at coarser spatial resolutions. At the vertex and parcel levels, however, these associations were not statistically significant when evaluated using spin-based spatial null models that accounted for spatial autocorrelation (both *p_spin_* > 0.106). Therefore, these findings should be interpreted as descriptive evidence of a large-scale cortical trend rather than as independent inferential evidence.

Together, these findings suggest a large-scale cortical trend in ASD-related INT differences rather than focal regional alterations.

### Individual deviations from the timescale hierarchy link sensory traits to cortical temporal dynamics

Finally, to examine whether individual variability in the cortical hierarchy of INTs is associated with behavioral traits, we analyzed the relationship between subject-specific deviations from the INT template and sensory processing profiles derived from the AASP questionnaire. Because the AASP indexes subjective sensory experience rather than primary sensory thresholds, it reflects integrative and regulatory aspects of sensory processing that extend beyond early sensory encoding (Dunn 1997, 2001). Such experiential dimensions may relate to variability in cortical temporal dynamics across hierarchical levels. Given that the large-scale hierarchical organization of INTs is preserved across individuals, inter-individual variability is expected to manifest primarily as deviations from this shared template rather than as alterations of the hierarchy itself. Quantifying subject-specific departures from the TDC-based INT template therefore provides a principled framework for characterizing individual variability while respecting the preserved hierarchical structure.

**Figure 5:**
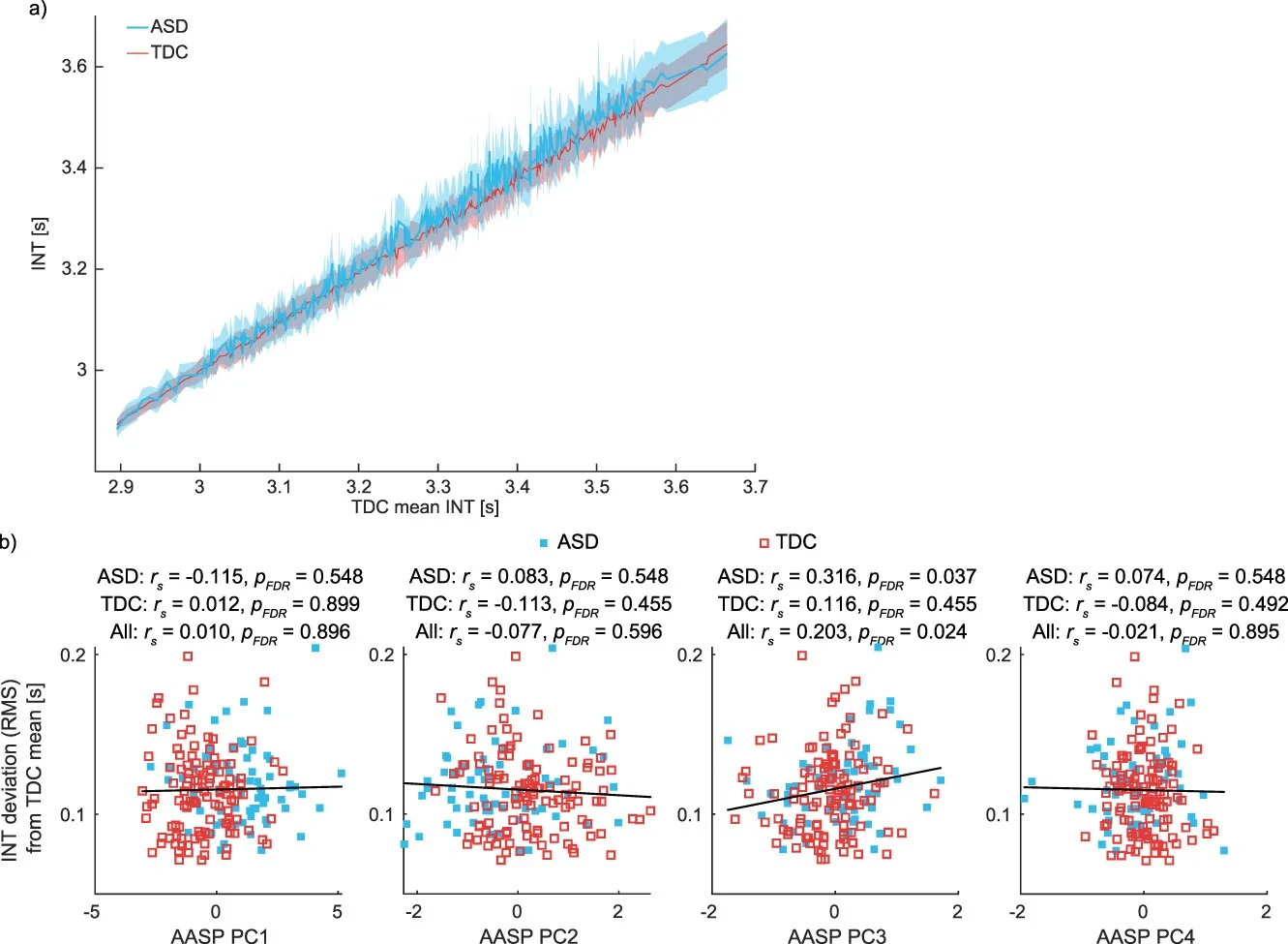
Individual deviations from the cortical hierarchy of intrinsic timescales and their relationship to sensory traits. (a) Group-averaged intrinsic neural timescale (INT) profiles plotted against the parcel-wise mean INT of the TDC group. Shaded areas represent 95% confidence intervals across participants. (b) Scatter plots showing relationships between individual deviation from the cortical timescale hierarchy, quantified as the root mean square (RMS) of residuals after linear alignment to the template, and principal component (PC) scores derived from the adolescent/adult sensory profile (AASP). PC1 reflects a general sensory reactivity dimension, PC2 is dominated by sensation seeking, PC3 contrasts low registration with sensation avoiding, and PC4 reflects opposing contributions of sensory sensitivity and sensation avoiding (see Table 2 for loadings). The numerical values displayed at the top of each panel indicate Spearman’s rank correlation coefficients (*r*_s_) and corresponding Benjamini–Hochberg false discovery rate-corrected *p* values across the four AASP PCs (*p*_FDR_).

As shown in Fig. 5a, group-averaged INT profiles plotted against the parcel-wise mean INT of the TDC group, used as a template, exhibited a highly similar monotonic relationship in both ASD and TDC groups, indicating preservation of the overall hierarchical organization of INTs. To formally assess this observation, we fitted a subject-wise linear model aligning individual INT profiles to the template and compared the resulting parameters across groups. The present dataset did not provide statistically significant evidence for group-related differences in either the global offset parameter (*α* in eq.(1); *p* = 0.145) or the scaling parameter (*β*; *p* = 0.147). These parameters capture overall prolongation or shortening of INTs and the strength of hierarchical alignment to the template, respectively. Meanwhile, these parameters were influenced by demographic and nuisance covariates, including age, sex, and head motion, underscoring the importance of accounting for such factors when characterizing individual INT profiles (Supplementary Table S4a, b).

After accounting for global offset and hierarchical scaling, the residual RMS measure did not show a significant association with diagnostic groups or other demographic variables (Supplementary Table S4c). Although the present dataset did not provide statistically significant evidence for a group-level effect, this does not imply that the residual variability is unstructured. Residual RMS values showed modest but statistically significant correspondence across independent run subsets in both ASD (Pearson’s *r* = 0.345, *p* = 0.004) and TDC (*r* = 0.367, *p* < 1 × 10^−5^), indicating that these deviations were not solely attributable to random scan-to-scan fluctuations. Rather, the residual RMS captures variance that was not significantly associated with by diagnostic or demographic variables examined here, making it well suited for probing continuous inter-individual differences that cut across diagnostic boundaries. To this end, we assessed relationships between the residual RMS measure and PC scores derived from the AASP, which were used to represent orthogonal sensory dimensions given the substantial correlations among AASP subscale raw scores, particularly among Low Registration, Sensory Sensitivity, and Sensation Avoiding (all *r_p_* > 0.60, p < 1 × 10^−5^). The PCA yielded four components with distinct loading patterns (Table 2): PC1 showed strong positive loadings on Low Registration, Sensory Sensitivity, and Sensation Avoiding, indicating a sensory modulation profile characterized by co-elevation across multiple response tendencies and elevated levels in ASD; PC2 was dominated by Sensation Seeking; PC3 exhibited a strong positive loading on Low Registration together with a substantial negative loading on Sensation Avoiding; PC4 was characterized by opposing contributions from Sensory Sensitivity and Sensation Avoiding. Correlation analyses were then performed between the residual RMS measure of INT and each AASP PC score.

**Table 2:** Group differences in adolescent/adult sensory profile (AASP) quadrant scores between individuals with autism spectrum disorder (ASD) and typically developed controls (TDC).

	ASD Mean (SD)	TDC Mean (SD)	Group difference	PC1 (61.210%)	PC2 (24.022%)	PC3 (9.257%)	PC4 (5.509%)
Low registration	39.388 (11.037)	26.947 (8.009)	*t*(178) = 8.553, *p* < 1 × 10^−5^	0.552	−0.084	0.798	−0.226
Sensation seeking	34.298 (9.459)	37.156 (9.025)	*t*(178) = -1.644, *p* = 0.101	0.182	0.976	−0.053	−0.107
Sensory sensitivity	40.925 (12.833)	31.669 (9.056)	*t*(178) = 5.535, *p* < 1 × 10^−5^	0.589	−0.034	−0.189	0.785
Sensation avoiding	42.209 (10.608)	32.191 (8.350)	*t*(178) = 6.790, *p* < 1 × 10^−5^	0.561	−0.198	−0.570	−0.567

Age and sex were included as covariates in the model to control for their potential confounding effects. Values in parentheses indicate the percentage of variance explained by each principal component (PC). Values in the PC columns represent component loadings.

As shown in Fig. 5b, partial correlation analyses controlling for age, sex, and FD did not provide statistically significant evidence for associations between INT residual RMS and PC1, PC2, or PC4 in either group or in the combined sample (all nominal *p* > 0.2). In contrast, a positive association emerged specifically for PC3. In the ASD group, higher PC3 scores were associated with larger residual RMS values (*r_s_* = 0.316, *p_FDR_* = 0.037 after BH-FDR correction across the four AASP PCs). A similar association was observed when all participants were analyzed together (*r_s_* = 0.203, *p_FDR_* = 0.024). Although the association was not statistically significant in the TDC group alone, the direction of the effect was consistent across groups. Similar results were obtained using the alternative AUC-based INT estimation procedure proposed by Watanabe et al. (2019) (Supplementary Table S5).

To assess the specificity of this effect, we conducted additional analyses using alternative INT metrics. First, we examined associations between raw INT values within the 12 functional networks and both AASP subscale scores and AASP PC scores. These exploratory analyses did not yield statistically significant correlations after BH-FDR correction in any group (Supplementary Tables S6 and S7). Second, we tested whether a simpler deviation metric–defined as the direct difference between individual INT profiles and the template, without decomposing global offset and hierarchical scaling components–was associated with AASP PC scores. No significant associations were observed (all *p_FDR_* > 0.250).

Taken together, these findings indicate that the observed behavioral association is not explained by raw INT magnitude or network-specific values per se, but rather emerges specifically from individual deviations from the TDC-based INT template after accounting for global offset and hierarchical scaling.

## Discussion

In this study, we investigated whether the hierarchical organization of INT—a straightforward index characterizing the temporal profile of fMRI signals—is altered in ASD, and how individual variation along this hierarchy relates to sensory-processing traits. By quantifying INTs across four five-minute resting-state runs and situating each individual along the

INT-based cortical hierarchy, we demonstrated that the overall hierarchical structure of INTs was preserved in ASD, while regions with longer INT exhibited further prolongation at the group level. Importantly, beyond global shifts and hierarchical scaling, we identified substantial individual-specific deviations from the canonical INT hierarchy. This pattern suggests that ASD is not characterized by a collapse of the hierarchical structure itself, but rather by systematic shifts and scaling biases that reflect demographic influences, alongside individual-specific deviations from this preserved template. These distributed deviations were selectively associated with sensory traits captured by the third PC of the AASP, characterized by high Low Registration and low Sensation Avoiding, indicating that variability in sensory processing is linked not to absolute INT values but to individual departures from the INT-based cortical hierarchy. Although some components of the residual variation may reflect measurement variability, individual deviation estimates showed modest but statistically significant split-half reliability across independent run subsets, indicating that the residual deviations were less stable than the hierarchy itself but were not solely attributable to random fluctuations across runs. The source of these residual deviations remains unclear; one possibility is that local perturbations within the hierarchy may manifest as distributed departures from the canonical profile. However, the current analysis does not allow these possibilities to be distinguished. Together, these findings indicate that while the macro-scale organization of cortical temporal dynamics remains largely intact in ASD, subtle individual-specific deviations—and their correspondence with sensory traits—reveal individual biases that may help explain heterogeneity in sensory experiences.

### Neurophysiological implications of prolonged timescales

A prolonged intrinsic timescale can be interpreted as reflecting stronger local recurrent interactions and reduced influence from rapidly fluctuating inputs. Computational and empirical studies have shown that longer timescales emerge when local circuits exhibit stronger recurrent excitation, increased temporal integration, or a shift in the excitation–inhibition balance toward excitation. These mechanisms have been repeatedly implicated in ASD, where altered local connectivity, elevated cortical excitability, and atypical inhibitory signaling have been documented across multiple levels of analysis, including cellular, circuit, and macroscopic imaging studies (Rubenstein and Merzenich 2003; Takarae and Sweeney 2017; Gogolla et al. 2009; Trakoshis et al. 2020). Viewed in this context, the prolongation observed in longer-timescale cortical regions in our sample aligns well with these prior accounts. Our findings therefore suggest that, despite the preservation of the overall cortical temporal hierarchy, individuals with ASD may exhibit subtle but systematic shifts in local circuit dynamics that bias certain regions toward slower intrinsic activity patterns. Such regional prolongation may reflect underlying circuit-level alterations that contribute to the sensory and cognitive characteristics associated with the condition.

### Discrepancy with reports indicating shortened INT in ASD

Several studies have reported locally shortened INTs in ASD (Watanabe et al. 2019; Uscătescu et al. 2023), whereas our findings indicate a largely preserved hierarchical organization of INTs at the group level. A key factor that may contribute to these discrepancies is the substantial heterogeneity within ASD, which increases the likelihood that individual studies—each sampling a different part of the spectrum—capture distinct patterns of cortical dynamics (Pelphrey et al. 2011; Lenroot and Yeung 2013; Tang et al. 2020). This issue is further amplified by sample-size–dependent variability: small samples are known to produce unstable estimates, including effects that can occasionally reverse in sign relative to the population-level pattern (Marek et al. 2022). To enhance the stability of intrinsic timescale estimation, we adopted multiple resting-state runs and conservative motion control criteria, both of which are expected to reduce measurement noise and improve the reliability of estimated INTs. However, the extent to which these procedures improve the accuracy of INT estimation relative to the underlying neural timescales, as well as the influence of alternative preprocessing choices, remains an important topic for future investigation.

Despite these efforts to improve estimation stability, larger samples would, in principle, further mitigate sampling fluctuations. However, expanding sample size in clinical neuroimaging is challenging. Data quality can be compromised by increased head motion, some individuals may decline or discontinue scanning due to sensory sensitivities, scanner noise, physical restraint, or anxiety, and comorbid psychiatric conditions (e.g., depression or panic symptoms) often limit eligibility for MRI participation. As a result, the present findings should be interpreted primarily in the context of ASD individuals who were able to complete relatively stable MRI acquisition under the present protocol and who could be meaningfully compared with TDC participants. This consideration may be particularly important in ASD, where exclusion based on head motion can systematically influence sample composition (Nebel et al. 2022). Future studies extending temporal-hierarchy analyses to broader ASD populations, including adolescents, older adults, and individuals with greater sensory or behavioral difficulties, will therefore require larger harmonized datasets, improved acquisition strategies, and ultimately meta-analytic integration. In particular, acquisition approaches that are more tolerant to motion and physiological artifacts, such as multi-echo fMRI, may be valuable.

Beyond sampling-related factors, it is also important to consider how INT should be interpreted as a signal-derived metric. Although INT is often discussed as reflecting the temporal window of neural information integration, it is formally estimated from measured neural signals and therefore depends not only on underlying neural dynamics but also on signal quality. Even if a neural system operates with relatively long intrinsic integration windows, increased noise, reduced signal-to-noise ratio, or motion-related artifacts can bias the estimated autocorrelation structure toward shorter timescales. Moreover, in the present study, because INT was estimated from blood oxygenation level-dependent (BOLD) fMRI signals, the resulting timescales may depend on physiological and vascular factors that influence the hemodynamic response. Consequently, variability in hemodynamic response properties across cortical regions and groups may contribute to estimated BOLD-derived timescales (Wu et al. 2021). Future large-scale studies incorporating acquisition and analysis strategies specifically designed for neural timescale estimation—such as multi-echo fMRI, hemodynamic calibration procedures, or hemodynamic response function deconvolution—will be valuable for further characterizing the contribution of vascular factors to estimated INTs. These considerations should be taken into account when interpreting BOLD-derived estimates of cortical temporal hierarchy in ASD. Complementary investigations using other measurement modalities, including electrocorticography and magnetoencephalography, may also help establish the robustness of cortical temporal hierarchies beyond the constraints of conventional fMRI acquisition.

In this sense, INT may be better understood as an index constrained by structural and physiological properties of cortical circuits, rather than a direct readout of functional integration timescales. While this makes INT valuable for characterizing large-scale hierarchical organization and inter-regional differences, caution is warranted when interpreting individual-level correlations or attributing changes in INT solely to alterations in cognitive or perceptual processing.

### Sensory traits relate to individual deviations from the cortical INT hierarchy

Beyond group-averaged effects, a central contribution of the present study lies in the characterization of individual-specific deviations from the INT-based cortical hierarchy. By explicitly modeling and removing global shifts and hierarchical scaling, we isolated distributed residual variability that captures how each individual departs from the canonical temporal organization of the cortex. This residual-based measure revealed meaningful associations with sensory traits, despite the absence of robust correlations between raw INT values and sensory measures at the level of individual networks. Together, these findings indicate that associations with sensory traits are not driven by absolute intrinsic timescale values at specific regions, but instead relate to distributed deviations from the INT-based cortical hierarchy. This observation is consistent with the view that sensory experience reflects coordinated processing across cortical regions rather than isolated regional dynamics (Uddin et al. 2013; Pessoa 2014, 2018).

The association between residual INT deviation and the AASP PC contrasting higher Low Registration with lower Sensation Avoiding underscores the relevance of a distributed perspective on sensory traits. This component captures a sensory profile characterized by reduced sensory registration accompanied by lower tendencies toward avoidance, independent of the shared variance among AASP subscales (largely reflected in PC1). The selective relationship with residual INT deviation suggests that such sensory profiles are linked not to absolute intrinsic timescale values in specific regions, but to how temporal processing is unevenly organized across the cortical hierarchy. In this context, increased deviation from the INT-based cortical hierarchy may reflect an altered balance between local temporal integration and hierarchical coordination, giving rise to idiosyncratic sensory experiences that are not well captured by group-level averages.

Importantly, the fact that this component reflects reduced sensory registration rather than heightened sensory sensitivity provides a plausible mechanistic link to temporal processing. Reduced sensory registration may require sensory evidence to be accumulated over longer temporal windows before reliably influencing perceptual experience (Wang 2002; Gold and Shadlen 2007), thereby rendering such profiles particularly sensitive to how temporal integration is distributed across cortical levels. By contrast, sensory hypersensitivity may arise from increased background activity or reduced signal-to-noise ratio in early sensory regions—mechanisms that may influence perceptual responsiveness without necessarily altering the large-scale distribution of temporal integration across cortical levels (Gerstner et al. 2014). Taken together, these considerations support the interpretation that residual deviations from the INT-based cortical hierarchy are consistent with alterations in temporal accumulation across processing levels, rather than general sensory responsiveness.

It is also worth noting that, while global offset and hierarchical scaling parameters did not provide statistically significant differences between diagnostic groups, they showed systematic associations with demographic factors. In particular, female participants exhibited significantly longer overall INTs and a shallower hierarchical slope (Supplementary Table S4a, b). However, given the relatively small number of female participants in the present sample, these effects should be interpreted cautiously and warrant further investigation in larger, sex-balanced cohorts.

More broadly, these results align with emerging views that emphasize ASD as a condition characterized not by a single canonical neural alteration, but by pronounced inter-individual variability in neural organization. From this perspective, deviations from normative hierarchical patterns—rather than shifts in the hierarchy itself—may constitute a key neural substrate of phenotypic heterogeneity. Residual-based metrics, such as those introduced here, offer a complementary approach to traditional group comparisons by directly quantifying individual differences in large-scale cortical dynamics, and may prove useful for linking intrinsic brain organization to behavioral diversity in both clinical and non-clinical populations.

### Limitations

Although no statistically significant difference was observed between the ASD and TDC groups in the auditory INT gradient, the direction of the effect was consistent with previous findings suggesting slightly reduced temporal integration in ASD (Watanabe et al. 2019; Uscătescu et al. 2023). Based on the observed group effect in the present data (Cohen’s *d* ≈ 0.21), detecting such a difference with 80% (90%) power would require approximately 233 (311) participants per group. More generally, increasing sample sizes remains an important consideration in neuroimaging studies to reliably characterize subtle group-level effects. At the same time, the small magnitude of the estimated group difference suggests that any potential alteration in auditory temporal hierarchy, if present, is likely modest. A further limitation concerns the substantial heterogeneity within the ASD group (Lenroot and Yeung 2013; Masi et al. 2017; Uljarević et al. 2017). Sensory-processing traits showed wide inter-individual variability, raising the possibility that subgroup-specific characteristics may influence the overall group estimates of INT, particularly in slow-timescale regions. Future studies with larger samples or stratification approaches may help clarify whether distinct sensory phenotypes exhibit different hierarchical signatures of INT. More broadly, such heterogeneity also raises the possibility that local or participant-specific variations in cortical organization may coexist with the preserved large-scale hierarchy observed at the group level.

Consistent with this point, the present analyses were performed using predefined canonical sensory hierarchies and fixed cortical parcellations in standard space, rather than subject-specific sensory topographies. Given previous evidence for substantial interindividual variability in the size and position of homologous functional regions (Li et al. 2019), the current framework cannot directly assess individual-specific areal boundaries or local remapping of hierarchical organization are preserved in ASD. Thus, the present findings should be interpreted as evidence for preservation of global cortical organization rather than preservation of fine-grained cortical topology.

Another important consideration is that the present study focused on temporal hierarchy derived from INTs. Recent studies have also proposed functional-connectivity–based gradient approaches as an alternative framework for characterizing cortical hierarchy (Nenning et al. 2020). These approaches quantify continuous spatial transitions in inter-regional connectivity structure and have emerged as a promising framework for describing large-scale cortical organization, including atypical cortical organization in ASD (Gao et al. 2025). The relationship between such functional gradients and temporal hierarchy derived from INT remains an important open question and should be examined in future studies.

Taken together, these considerations highlight the need for future work that integrates larger samples and multimodal neural markers to more precisely characterize how cortical temporal hierarchies vary across individuals and contribute to sensory experiences.

## Supplementary Material

Document_int_asd_jul202026_supp

## Data Availability

The imaging data analyzed in this study were obtained from the Brain/MINDS Beyond Human Brain MRI Project. Access to the dataset is available upon request through the Brain/MINDS Beyond data-sharing program at https://hbm.brainminds-beyond.jp/data.html. To facilitate reproducibility of the analyses developed for the present study, the MATLAB code used for spin-based spatial null testing for sensory hierarchy analyses, template-based hierarchical modeling, estimation of individual deviation metrics, and statistical analyses is publicly available at https://github.com/shikauchiy/int-hierarchy-analysis.
