# Supplementary material for: Preserved intrinsic neural timescale organization with hierarchical variation in autism spectrum disorder
Source: Cereb Cortex. 2026 Sep 7;36(9):bhag121. doi: 10.1093/cercor/bhag121 (PMC13548320; doi:10.1093/cercor/bhag121)
Supplement: Supplementary file 1 [file document_int_asd_jul202026_supp.docx]

Supplementary Materials

Preserved Intrinsic Neural Timescale Organization with Hierarchical Variation in Autism Spectrum Disorder

*Yumi Shikauchi^1^, *Ryuta Aoki^1,2,3^, Takashi Itahashi^1^, Masaaki Shimizu^4^, Taiga Naoe^1^, Tsukasa Okimura^1^, Haruhisa Ohta^1^, Ryu-ichiro Hashimoto^1,3^, Motoaki Nakamura^1^

1. Medical Institute of Developmental Disabilities Research, Showa Medical University, Tokyo 157-8577, Japan

2. Graduate School of Medicine, Human Brain Research Center, Kyoto University, kyoto 606-8507, Japan

3. Department of Language Sciences, Graduate School of Humanities, Tokyo Metropolitan University, Tokyo 192-0397, Japan

4. Graduate School of Medical and Dental Sciences, Institute of Science Tokyo, Tokyo 113-8510, Japan

Corresponding author: Motoaki Nakamura, MD., PhD. [motoaki@med.showa-u.ac.jp](mailto:motoaki@med.showa-u.ac.jp)

6-11-11 Kitakarasuyama, Setagaya-ku, Tokyo 157-8577, Japan

*: Co-first author

Supplementary Methods

Split-half robustness analysis

To evaluate whether preservation of the cortical INT hierarchy depended on averaging across all four resting-state runs, we additionally performed split-half robustness analyses using independent run subsets. Because resting-state fMRI acquisition was performed with alternating anterior-to-posterior and posterior-to-anterior phase-encoding directions across runs, INT maps were separately estimated from the first two runs and the last two runs for both ASD and TDC groups. Spatial correspondence analyses between ASD and TDC were then repeated independently for each split-half dataset at the vertex-, parcel-, and network-wise levels.

In addition, individual residual RMS values were estimated separately for each split-half dataset, and split-half reliability was assessed using Pearson correlation across participants.

FD-matched sensitivity analysis

To evaluate the robustness of main findings to differences in head motion, we additionally performed FD-matched sensitivity analyses. ASD participants were retained unchanged, and a TDC subsample was generated using nearest-neighbor matching based on mean FD, without replacement. Specifically, for each ASD participant, the TDC participant with the closest mean FD value was selected. The resulting matched sample showed no significant group difference in mean FD (matched TDC: mean ± SD = 0.0720 ± 0.0262 mm; ASD: 0.0729 ± 0.0274 mm; Mann–Whitney U test, *p* = 0.904). Spatial correspondence analyses of cortical INT hierarchy were then repeated using the FD-matched TDC sample.

Supplementary Tables and Figures

**Supplementary Table S1. Sensitivity analysis of sensory hierarchy analyses using the area-under-curve-based intrinsic neural timescale (INT) definition proposed by** [**Watanabe et al. (2019**](https://paperpile.com/c/1qQ4j7/Wbh5)**).** Participant-level correspondence between regional INTs and predefined sensory hierarchies was quantified using Spearman’s correlation coefficient (*r_s_*). Group effects and Cohen’s *d* values were estimated from linear models controlling for age, sex, and mean framewise displacement.

| Sensory System | ASD median *r_s_* [IQR] | TDC median *r_s_* [IQR] | Group effect *p* | Cohen’s *d* |
| --- | --- | --- | --- | --- |
| Auditory | 0.783 [0.246] | 0.817 [0.200] | 0.074 | 0.282 |
| Visual | 0.283 [0.654] | 0.367 [0.546] | 0.569 | 0.089 |
| Somatosensory | 0.600 [0.387] | 0.567 [0.375] | 0.618 | -0.078 |

**Supplementary Table S2. Split-half reliability of sensory intrinsic neural timescale (INT) hierarchies estimated from independent run subsets.** Reliability was quantified as the within-subject Spearman correlation (*r_s_*) between sensory INT profiles estimated from the first two and last two resting-state runs. Group effects were assessed using linear models including age, sex, and mean framewise displacement as covariates.

| Sensory system | ASD median *r_s_* [IQR] | TDC median *r_s_* [IQR] | Group effect p |
| --- | --- | --- | --- |
| Auditory | 0.817 [0.292] | 0.800 [0.212] | 0.533 |
| Visual | 0.450 [0.362] | 0.533 [0.462] | 0.402 |
| Somatosensory | 0.683 [0.442] | 0.700 [0.458] | 0.612 |

**Supplementary Figure S1. Preserved cortical hierarchy of intrinsic neural timescales (INTs) in autism spectrum disorder (ASD) after framewise displacement (FD)-matched sensitivity analysis.**

Scatter plots illustrate the correspondence of vertex- (a), parcel- (b), and network-wise (c) INTs between ASD participants and an FD-matched subsample of typically developed controls (TDC). Each dot represents a cortical vertex, parcel, or functional network, and the gray diagonal line indicates perfect correspondence (Pearson’s *r_p_* = 1) between ASD and the FD-matched TDC sample. In the parcel-wise plot, colors correspond to cortical regions defined in the Glasser multimodal parcellation [(Glasser et al. 2016)](https://paperpile.com/c/1qQ4j7/ZYU8V), whereas in the network-wise plot, colors correspond to canonical large-scale functional networks based on the Cole–Anticevic brain-wide network partition [(Ji et al. 2019)](https://paperpile.com/c/1qQ4j7/3eby).

**Supplementary Table S3. Parcels showing nominal between-group differences in intrinsic neural timescale identified by ANCOVA (autism spectrum disorder [ASD] versus typically developed controls [TDC]), with age, sex and mean fra	mewise displacement included as covariates.** No parcels remained statistically significant after Benjamini–Hochberg false discovery rate correction across parcels. R, right; L, left; PIT, posterior inferior temporal area; LO2, lateral occipital area 2; MIP, medial intraparietal area; 7PC, part of Broadman area 7 in the superior parietal cortex; a9-46v, anterior 9-46 ventral; V3CD, visual area V3 complex dorsal subdivision; VIP, ventral intraparietal area; MT, middle temporal area; V4, visual area V4.

| Parcel name | Network label | *p* value (unc.) | *p* value  (FDR) | ASD | TDC | Cohen’s *d* |
| --- | --- | --- | --- | --- | --- | --- |
| R_PIT | Visual2 | 0.007 | 0.822 | 3.531 | 3.417 | -0.420 |
| R_LO2 | Visual2 | 0.015 | 0.822 | 3.611 | 3.502 | -0.378 |
| L_MIP | Dorsal Attention | 0.022 | 0.822 | 3.560 | 3.478 | -0.355 |
| L_7PC | Somatomotor | 0.030 | 0.822 | 3.429 | 3.342 | -0.336 |
| L_a9-46v | Frontoparietal | 0.032 | 0.822 | 3.499 | 3.429 | -0.333 |
| R_V3CD | Visual2 | 0.035 | 0.822 | 3.637 | 3.548 | -0.327 |
| L_VIP | Visual2 | 0.036 | 0.822 | 3.516 | 3.434 | -0.324 |
| L_MT | Visual2 | 0.041 | 0.822 | 3.456 | 3.365 | -0.316 |
| R_V4 | Visual2 | 0.047 | 0.822 | 3.510 | 3.443 | -0.308 |

**Supplementary Figure S2. Spatial distribution of parcels showing group-related divergence in intrinsic neural timescales**

Binary cortical surface maps illustrating the anatomical locations of Glasser parcels showing nominal group differences between the ASD and TDC groups. Parcels with uncorrected *p* < 0.05 in the parcel-wise comparison are highlighted in yellow, whereas all other parcels are shown in gray.

**Supplementary Table S4. Effects of diagnostic group and covariates on individual deviation metrics derived from intrinsic neural timescale (INT) profiles.**

ANCOVA models included group (ASD vs. TDC), age, sex, and mean framewise displacement (FD) as predictors. The global offset parameter (<https://www.codecogs.com/eqnedit.php?latex=%5Calpha#0>) reflects overall prolongation or shortening of INTs (a), the scaling parameter (<https://www.codecogs.com/eqnedit.php?latex=%5Cbeta#0>) reflects hierarchical alignment to the template (b), and<https://www.codecogs.com/eqnedit.php?latex=%5Cmathrm%7Brms%7D#0> quantifies individual-specific deviations from the INT-based cortical hierarchy after accounting for global and hierarchical effects (c). Significant effects (*p* < 0.05) are shown in bold.

a) Global offset parameter (<https://www.codecogs.com/eqnedit.php?latex=%5Calpha#0>)

Model statistics: *F*(4,177) = 8.633, *p* < 1 × 10^-5^

| Predictor | Estimate | SE | *t* | *p* |
| --- | --- | --- | --- | --- |
| Group (TDC) | 0.176 | 0.120 | 1.460 | 0.145 |
| **Age** | **-0.017** | **0.008** | **-2.070** | **0.040** |
| **Sex (Female)** | **0.623** | **0.130** | **4.810** | **< 1 × 10^-5^** |
| **FD** | **4.957** | **2.281** | **2.170** | **0.031** |

b) Hierarchical scaling parameter (<https://www.codecogs.com/eqnedit.php?latex=%5Cbeta#0>)

Model statistics: *F*(4,177) = 7.329, *p* = 1.744 × 10^-5^

| Predictor | Estimate | SE | *t* | *p* |
| --- | --- | --- | --- | --- |
| Group (TDC) | -0.062 | 0.042 | -1.456 | 0.147 |
| Age | 0.004 | 0.003 | 1.436 | 0.153 |
| **Sex (Female)** | **-0.212** | **0.046** | **-4.634** | **< 1 × 10^-5^** |
| FD | -1.499 | 0.805 | -1.864 | 0.064 |

c) Residual deviation from template (<https://www.codecogs.com/eqnedit.php?latex=%5Cmathrm%7Brms%7D#0>)

Model statistics: *F*(4,177) = 0.828, *p* = 0.508

| Predictor | Estimate | SE | *t* | *p* |
| --- | --- | --- | --- | --- |
| Group (TDC) | -0.006 | 0.004 | -1.570 | 0.119 |
| Age | 0.000 | 0.000 | 0.400 | 0.690 |
| Sex (Female) | -0.003 | 0.004 | -0.750 | 0.453 |
| FD | -0.048 | 0.077 | -0.620 | 0.535 |

**Supplementary Table S5. Associations between residual deviations from the area-under-curve-based intrinsic neural timescale (INT) hierarchy and Adolescent/Adult Sensory Profile (AASP) principal component scores.**

Partial Spearman correlations between residual root mean square (RMS) deviations from the template hierarchy derived using the alternative INT estimation procedure proposed by [Watanabe et al. (2019)](https://paperpile.com/c/1qQ4j7/Wbh5) and AASP principal component (PC) scores. Correlations were adjusted for age, sex, and framewise displacement. *P*-values were corrected across the four AASP PCs using the Benjamini–Hochberg false discovery rate procedure. Significant associations (*p*_FDR_ < 0.05) are shown in bold.

|  | PC1 | | PC2 | | PC3 | | PC4 | |
| --- | --- | --- | --- | --- | --- | --- | --- | --- |
|  | *r_s_* | *p*_FDR_ | *r_s_* | *p*_FDR_ | *r_s_* | *p*_FDR_ | *r_s_* | *p*_FDR_ |
| ASD | -0.122 | 0.434 | 0.137 | 0.434 | **0.326** | **0.029** | 0.015 | 0.902 |
| TDC | 0.033 | 0.742 | -0.151 | 0.429 | 0.099 | 0.589 | -0.031 | 0.742 |
| All | 0.030 | 0.874 | -0.076 | 0.611 | **0.195** | **0.034** | -0.012 | 0.874 |

**Supplementary Table S6. Spearman correlations between intrinsic neural timescales (INT) and Adolescent/Adult Sensory Profile (AASP) subscale scores across functional networks.**

Spearman’s rank correlation coefficients (*r_s_*), uncorrected p-values (*p_unc_*) are reported for each AASP subscale (Low Registration, Sensation Seeking, Sensory Sensitivity, Sensation Avoiding) within each functional network separately for ASD, TDC, and the combined participant (All). Cells in bold indicate associations with uncorrected *p* < 0.05. No association significant after Benjamini–Hochberg false discovery rate correction across four subscores (all > 0.700).

|  |  | Low Registration | | Sensation Seeking | | Sensory Sensitivity | | Sensation Avoiding | |
| --- | --- | --- | --- | --- | --- | --- | --- | --- | --- |
|  |  | *r_s_* | *p* | *r_s_* | *p* | *r_s_* | *p* | *r_s_* | *p* |
| Visual | ASD | 0.083 | 0.504 | 0.073 | 0.555 | -0.025 | 0.842 | -0.097 | 0.437 |
|  | TDC | 0.047 | 0.620 | -0.166 | 0.077 | 0.028 | 0.770 | 0.062 | 0.507 |
|  | All | 0.097 | 0.194 | -0.073 | 0.328 | 0.019 | 0.794 | 0.025 | 0.733 |
| Visual2 | ASD | 0.000 | 0.997 | 0.089 | 0.476 | -0.091 | 0.462 | -0.136 | 0.272 |
|  | TDC | 0.065 | 0.492 | -0.174 | 0.063 | 0.013 | 0.889 | 0.030 | 0.749 |
|  | All | 0.084 | 0.257 | -0.076 | 0.311 | -0.004 | 0.961 | 0.004 | 0.958 |
| Somatomotor | ASD | 0.018 | 0.883 | 0.102 | 0.410 | -0.127 | 0.306 | -0.208 | 0.092 |
|  | TDC | 0.027 | 0.779 | -0.116 | 0.216 | 0.000 | 0.997 | -0.054 | 0.568 |
|  | All | 0.061 | 0.414 | -0.031 | 0.676 | -0.024 | 0.752 | -0.080 | 0.282 |
| Cingulo-Opercular | ASD | 0.076 | 0.539 | 0.054 | 0.664 | -0.090 | 0.469 | -0.122 | 0.327 |
|  | TDC | 0.044 | 0.642 | -0.122 | 0.194 | 0.057 | 0.545 | -0.002 | 0.979 |
|  | All | 0.067 | 0.371 | -0.052 | 0.482 | 0.006 | 0.937 | -0.038 | 0.614 |
| Dorsal Attention | ASD | 0.044 | 0.725 | 0.146 | 0.239 | -0.092 | 0.460 | -0.122 | 0.325 |
|  | TDC | 0.022 | 0.815 | -0.158 | 0.092 | 0.037 | 0.697 | -0.009 | 0.920 |
|  | All | 0.065 | 0.383 | -0.048 | 0.522 | 0.012 | 0.876 | -0.018 | 0.812 |
| Language | ASD | 0.046 | 0.712 | 0.045 | 0.720 | -0.117 | 0.344 | -0.157 | 0.204 |
|  | TDC | 0.056 | 0.551 | -0.137 | 0.144 | 0.093 | 0.322 | 0.079 | 0.399 |
|  | All | 0.074 | 0.323 | -0.062 | 0.405 | 0.025 | 0.738 | 0.008 | 0.920 |
| Frontoparietal | ASD | 0.079 | 0.527 | 0.076 | 0.543 | -0.104 | 0.401 | -0.088 | 0.479 |
|  | TDC | 0.021 | 0.827 | -0.154 | 0.099 | 0.052 | 0.583 | 0.049 | 0.605 |
|  | All | 0.062 | 0.407 | -0.063 | 0.395 | 0.003 | 0.973 | 0.010 | 0.894 |
| Auditory | ASD | -0.002 | 0.988 | 0.054 | 0.663 | -0.154 | 0.213 | **-0.251** | **0.041** |
|  | TDC | 0.044 | 0.639 | -0.160 | 0.088 | 0.043 | 0.647 | -0.023 | 0.806 |
|  | All | 0.054 | 0.470 | -0.069 | 0.358 | -0.024 | 0.748 | -0.081 | 0.279 |
| Default | ASD | 0.067 | 0.588 | 0.094 | 0.451 | -0.110 | 0.375 | -0.071 | 0.568 |
|  | TDC | 0.053 | 0.573 | -0.122 | 0.193 | 0.073 | 0.438 | 0.041 | 0.665 |
|  | All | 0.061 | 0.411 | -0.028 | 0.705 | 0.004 | 0.961 | -0.002 | 0.981 |
| Posterior Multimodal | ASD | 0.016 | 0.896 | 0.060 | 0.629 | -0.110 | 0.376 | -0.184 | 0.135 |
|  | TDC | 0.047 | 0.620 | -0.150 | 0.109 | 0.040 | 0.669 | -0.001 | 0.989 |
|  | All | 0.075 | 0.317 | -0.072 | 0.337 | -0.001 | 0.988 | -0.038 | 0.609 |
| Ventral Multimodal | ASD | 0.087 | 0.483 | 0.048 | 0.697 | -0.001 | 0.996 | -0.114 | 0.360 |
|  | TDC | 0.082 | 0.384 | -0.139 | 0.138 | 0.055 | 0.563 | 0.037 | 0.693 |
|  | All | 0.113 | 0.129 | -0.069 | 0.356 | 0.045 | 0.545 | -0.002 | 0.977 |
| Orbito-Affective | ASD | 0.024 | 0.847 | 0.045 | 0.720 | -0.056 | 0.655 | -0.127 | 0.307 |
|  | TDC | 0.110 | 0.244 | -0.140 | 0.136 | 0.070 | 0.460 | 0.026 | 0.782 |
|  | All | 0.059 | 0.428 | -0.055 | 0.459 | -0.006 | 0.932 | -0.061 | 0.415 |

**Supplementary Table S7. Spearman correlations between intrinsic neural timescales (INT) and principal component (PC) scores derived from the Adolescent/Adult Sensory Profile (AASP) across functional networks.**

Spearman’s rank correlation coefficients (*r_s_*) and uncorrected *p*-values are shown for each AASP PC across functional networks, analyzed separately in ASD, TDC, and the combined participant sample (All). PC analysis was applied to the four AASP subscales to obtain orthogonal sensory dimensions capturing shared and distinct variance across subscales. Cells in bold indicate associations with uncorrected p < 0.05. No associations remained significant after Benjamini–Hochberg false discovery rate correction across four PCs (all > 0.08).

|  |  | PC1 | | PC2 | | PC3 | | PC4 | |
| --- | --- | --- | --- | --- | --- | --- | --- | --- | --- |
|  |  | *r_s_* | *p* | *r_s_* | *p* | *r_s_* | *p* | *r_s_* | *p* |
| Visual | ASD | -0.003 | 0.981 | 0.073 | 0.558 | **0.315** | **0.010** | 0.092 | 0.458 |
|  | TDC | 0.015 | 0.874 | **-0.197** | **0.035** | 0.038 | 0.684 | 0.022 | 0.815 |
|  | All | 0.035 | 0.638 | -0.103 | 0.168 | **0.160** | **0.032** | 0.029 | 0.696 |
| Visual2 | ASD | -0.070 | 0.571 | 0.116 | 0.350 | **0.259** | **0.034** | 0.056 | 0.652 |
|  | TDC | 0.009 | 0.927 | **-0.199** | **0.033** | 0.097 | 0.304 | 0.007 | 0.941 |
|  | All | 0.021 | 0.781 | -0.095 | 0.201 | **0.166** | **0.025** | 0.015 | 0.842 |
| Somatomotor | ASD | -0.109 | 0.379 | 0.149 | 0.229 | **0.337** | **0.006** | 0.080 | 0.519 |
|  | TDC | -0.027 | 0.770 | -0.121 | 0.199 | 0.114 | 0.224 | 0.108 | 0.251 |
|  | All | -0.019 | 0.801 | -0.021 | 0.781 | **0.201** | **0.007** | 0.092 | 0.217 |
| Cingulo-Opercular | ASD | -0.059 | 0.636 | 0.061 | 0.622 | **0.296** | **0.015** | 0.035 | 0.779 |
|  | TDC | 0.021 | 0.827 | -0.141 | 0.133 | 0.097 | 0.304 | 0.128 | 0.173 |
|  | All | 0.009 | 0.906 | -0.057 | 0.442 | **0.159** | **0.032** | 0.095 | 0.202 |
| Dorsal Attention | ASD | -0.045 | 0.715 | 0.161 | 0.193 | **0.294** | **0.016** | 0.038 | 0.760 |
|  | TDC | -0.002 | 0.983 | -0.174 | 0.063 | 0.087 | 0.355 | 0.106 | 0.258 |
|  | All | 0.020 | 0.790 | -0.057 | 0.441 | **0.158** | **0.033** | 0.078 | 0.297 |
| Language | ASD | -0.077 | 0.537 | 0.060 | 0.628 | **0.323** | **0.008** | 0.048 | 0.698 |
|  | TDC | 0.071 | 0.453 | -0.168 | 0.073 | 0.025 | 0.790 | 0.081 | 0.391 |
|  | All | 0.037 | 0.622 | -0.075 | 0.314 | 0.135 | 0.069 | 0.067 | 0.368 |
| Frontoparietal | ASD | -0.033 | 0.790 | 0.081 | 0.512 | **0.282** | **0.021** | -0.009 | 0.942 |
|  | TDC | 0.023 | 0.810 | -0.182 | 0.052 | 0.032 | 0.737 | 0.059 | 0.530 |
|  | All | 0.021 | 0.780 | -0.078 | 0.295 | 0.130 | 0.081 | 0.032 | 0.664 |
| Auditory | ASD | -0.144 | 0.245 | 0.122 | 0.323 | **0.355** | **0.003** | 0.045 | 0.719 |
|  | TDC | -0.004 | 0.967 | -0.173 | 0.065 | 0.106 | 0.259 | 0.144 | 0.124 |
|  | All | -0.029 | 0.695 | -0.054 | 0.469 | **0.192** | **0.010** | 0.096 | 0.197 |
| Default | ASD | -0.031 | 0.800 | 0.089 | 0.474 | **0.264** | **0.031** | -0.056 | 0.653 |
|  | TDC | 0.039 | 0.677 | -0.144 | 0.125 | 0.053 | 0.574 | 0.072 | 0.442 |
|  | All | 0.017 | 0.825 | -0.039 | 0.600 | 0.129 | 0.083 | 0.027 | 0.718 |
| Posterior Multimodal | ASD | -0.097 | 0.435 | 0.099 | 0.423 | **0.338** | **0.005** | 0.050 | 0.688 |
|  | TDC | 0.019 | 0.841 | -0.170 | 0.069 | 0.101 | 0.282 | 0.088 | 0.351 |
|  | All | 0.008 | 0.914 | -0.073 | 0.329 | **0.186** | **0.012** | 0.068 | 0.364 |
| Ventral Multimodal | ASD | 0.006 | 0.959 | 0.069 | 0.576 | **0.306** | **0.012** | 0.140 | 0.257 |
|  | TDC | 0.041 | 0.661 | -0.169 | 0.070 | 0.081 | 0.387 | 0.067 | 0.479 |
|  | All | 0.048 | 0.517 | -0.082 | 0.271 | **0.169** | **0.023** | 0.089 | 0.233 |
| Orbito-Affective | ASD | -0.045 | 0.715 | 0.070 | 0.575 | **0.256** | **0.037** | 0.106 | 0.395 |
|  | TDC | 0.044 | 0.642 | -0.161 | 0.086 | 0.114 | 0.225 | 0.100 | 0.286 |
|  | All | -0.015 | 0.840 | -0.047 | 0.525 | **0.164** | **0.027** | 0.121 | 0.105 |
